# Associations of supermarket accessibility with obesity and fruit and vegetable consumption in the conterminous United States

**DOI:** 10.1186/1476-072X-9-49

**Published:** 2010-10-08

**Authors:** Akihiko Michimi, Michael C Wimberly

**Affiliations:** 1 Geographic Information Science Center of Excellence, South Dakota State University, Wecota Hall Box 506B, 1021 Medary Avenue, Brookings, SD 57007, USA

## Abstract

**Background:**

Limited access to supermarkets may reduce consumption of healthy foods, resulting in poor nutrition and increased prevalence of obesity. Most studies have focused on accessibility of supermarkets in specific urban settings or localized rural communities. Less is known, however, about how supermarket accessibility is associated with obesity and healthy diet at the national level and how these associations differ in urban versus rural settings. We analyzed data on obesity and fruit and vegetable (F/V) consumption from the Behavioral Risk Factor Surveillance System for 2000-2006 at the county level. We used 2006 Census Zip Code Business Patterns data to compute population-weighted mean distance to supermarket at the county level for different sizes of supermarket. Multilevel logistic regression models were developed to test whether population-weighted mean distance to supermarket was associated with both obesity and F/V consumption and to determine whether these relationships varied for urban (metropolitan) versus rural (nonmetropolitan) areas.

**Results:**

Distance to supermarket was greater in nonmetropolitan than in metropolitan areas. The odds of obesity increased and odds of consuming F/V five times or more per day decreased as distance to supermarket increased in metropolitan areas for most store size categories. In nonmetropolitan areas, however, distance to supermarket had no associations with obesity or F/V consumption for all supermarket size categories.

**Conclusions:**

Obesity prevalence increased and F/V consumption decreased with increasing distance to supermarket in metropolitan areas, but not in nonmetropolitan areas. These results suggest that there may be a threshold distance in nonmetropolitan areas beyond which distance to supermarket no longer impacts obesity and F/V consumption. In addition, obesity and food environments in nonmetropolitan areas are likely driven by a more complex set of social, cultural, and physical factors than a single measure of supermarket accessibility. Future research should attempt to more precisely quantify the availability and affordability of foods in nonmetropolitan areas and consider alternative sources of healthy foods besides supermarkets.

## Background

The prevalence of obesity is a growing health concern for children, adolescents, and adults in the United States and other countries. According to the Dietary Guidelines for Americans 2005, poor diet and physical inactivity, resulting in an energy imbalance, are the most important factors contributing to the increase in overweight and obesity in the United States [1]. Although the obesity epidemic is widespread within the United States, obesity has more seriously affected specific subgroup populations and geographic areas. For example, the prevalence of overweight and obesity is generally higher in rural areas than in urban areas, particularly among African Americans [2-4]. Yet, geographic distributions of obesity and associated risk factors are spatially heterogeneous, which suggests that obesity prevalence is not higher in all rural areas, and likewise, that prevalence is not lower in all urban areas [2,5].

Much obesity research in the developed world has addressed the issue of food deserts and the influences of neighborhood environments on obesity. A food desert is defined as an area with limited access to affordable and nutritious food, particularly in lower income communities [6]. Reduced consumption of healthy foods, such as fruit and vegetables, and increased consumption of energy-dense foods in these areas may cause poor nutrition, increased obesity, and increased prevalence of chronic diseases such as type 2 diabetes [7,8]. Obesity and healthy food consumption are influenced by multiple aspects of the environment, including economic, sociocultural, and physical factors [9-12]. It is also important to recognize that populations in food deserts of the United States can be identified at a variety of spatial scales ranging from neighborhood to regional levels [13,14].

Recent literature on obesity and the food environment has suggested that obesity risk decreases as people live closer to supermarkets rather than convenience stores [15-17]. In addition, median household income is associated with greater healthy food availability, and larger stores have lower prices for healthy foods [18]. Economically deprived areas and lower income neighborhoods have fewer stores and are located further away from the nearest supermarkets carrying fresh produce [14,19,20]. These neighborhoods have more fast food retail outlets, liquor stores, and convenience stores [21,22]. On the contrary, limited geographic availability of supermarkets was not associated with obesity risk among low-income women in Kansas [23]. The food environment can affect health in both disadvantaged inner-city urban areas and remote rural communities, but is likely to have different causes and consequences within each of these distinct environments [24].

### Urban food deserts

In urban areas, people often have unequal access to food retailers that carry healthy food because of their socioeconomic status and residential location. Impoverished neighborhoods, predominantly occupied by minority groups, were further away from the nearest supermarket than were wealthier neighborhoods in large metropolitan areas, such as Detroit [20], Los Angeles [19], and London, Ontario [25]. In addition, the food environment was generally less diverse and lower quality in poor and minority neighborhoods than in wealthier and predominantly white neighborhoods of urban counties in Maryland, North Carolina, and New York [22] and in New Haven, Connecticut [26]. In Tunisia, people who frequently shopped at supermarkets had a slightly improved diet quality, compared to those who shopped at other smaller retail outlets [27].

In contrast, fast-food restaurants and convenience stores were concentrated within walking distances from schools in deprived neighborhoods of Chicago [28] and East Los Angeles [29] and were close to residents with lower socioeconomic status [21,30,31] which suggests that children and low-income residents have greater access to poor quality food in urban areas. Energy-dense foods were not only less expensive than healthier foods, but also more resistant to inflation, resulting in the highest rates of obesity among residents of limited economic means [32]. Therefore, pricing interventions of taxes on energy-dense food, subsidies for the purchase of healthy food, and improved economic and physical access to supermarket may be essential for health promotion [33]. These studies suggest that people in disadvantaged urban communities are forced to travel longer distance to reach food retailers that carry affordable healthy foods, compared to people living in wealthier neighborhoods, where supermarkets are more abundant.

### Rural food deserts

Rural food deserts, in contrast, have aspects that are fundamentally different from urban food deserts. Rural neighborhoods have fewer chain supermarkets than urban areas and have poor geographic access to supermarkets and healthy foods [31]. Rural areas offer fewer public transportation services than in urban areas, and this lack of transportation infrastructure is the most defining characteristics in rural communities with limited food access [6]. Large food retailers are often limited to larger towns, resulting in greater travel distances to reach services among isolated rural residents, such as in the Lower Mississippi Delta [34] as well as in rural Georgia [9]. In parts of rural Texas, neighborhoods were further away from supermarkets or full-line grocery stores and closer to convenience stores, although some of the most deprived neighborhoods had better access to the nontraditional food stores that sold a variety of fruit and vegetables [35,36]. Yet, the types of food stores available and the range of healthy foods offered vary greatly across diverse low-income communities [37]. In addition, food consumption patterns were associated with poverty levels in rural communities, indicating that the diet quality was lower among adults with insufficient food supply [38]. This may be in part due to the fact that convenience stores and smaller grocery stores that are common in many rural communities generally have lower availability of healthy foods at higher costs than supermarkets [6,39].

From an economic perspective, over the past 30 years the restructuring of food retail industries has occurred such that local grocery stores that once served small rural communities have been closed and replaced by or consolidated into a few regional and national chain grocers and supercenters [40]. As a result of this restructuring, remote rural residents must travel longer distances to reach a few large food retailers, which generally carry a wide variety of healthy food items at a relatively low cost. This uneven distribution of large food retailers creates problems of food deserts in rural communities. Rural residents with lower income, particularly younger age groups, are more likely to be food insecure than other rural populations [41]. With regard to geographic regions, the Great Plains are especially lacking in easy access to grocery stores [42].

To date, most studies have focused on the accessibility of large supermarkets that carry a large assortment of fresh fruit and vegetables and have been conducted in specific urban areas [15,19,20,43,44]. Other studies in urban environments have documented the association of obesity with neighborhood deprivation and access to fast food retailers [30,45] or examined the spatial patterns of both supermarkets and fast food and/or convenience store locations in the context of nutrition and obesity [15,21,22,29]. Studies of food environments in rural areas have become more common recently, and they have also focused on specific local communities and regions, [9,24,34-38,41]. As a result, it is difficult to generalize their results beyond the local study areas.

There is some evidence that people with no supermarkets near their homes are less likely to have a healthy diet than those with the most stores [46]. However, there is a need for more information about how supermarket accessibility is associated with obesity and healthy food consumption nationwide, and how distance to supermarket is related to obesity prevalence and fruit and vegetable consumption in both urban and rural areas. This research examines geographic patterns of supermarket accessibility at the national level by mapping population-weighted mean distance to supermarket for every county in the conterminous United States. The associations of distance to supermarket with obesity and fruit and vegetable consumption are then investigated by linking the supermarket accessibility data with health surveillance data from the Behavioral Risk Factor Surveillance System (BRFSS). Although the term 'food desert' is multifaceted in its usage, this research focuses on distance as a physical constraint that may limit access to healthy food. Based on the existing literature, we test two hypotheses: (1) the population-weighted mean distances to supermarket are positively related to obesity prevalence and negatively related to fruit and vegetable consumption at the national level and (2) the associations of obesity and fruit and vegetable consumption with distance to supermarket vary between metropolitan and nonmetropolitan counties, reflecting the unique characteristics of urban versus rural food environments.

## Methods

### Data

Data on obesity and fruit and vegetable (F/V) consumption from 2000 to 2006 were obtained from the BRFSS. Established in 1984 by the Centers for Disease Control and Prevention (CDC), BRFSS is an annual cross-sectional telephone survey that provides state-specific data on health risk behaviors and other information related to chronic diseases. The BRFSS uses random digit dialing and collects data from non-institutionalized civilian adults aged over 18 years. We requested non-public use BRFSS data via a written request to the CDC, in which a county FIPS code was assigned to all individual records. Sample weights were provided to adjust for the differences in probability of sample selection and sample design weights were used for deriving representative population-based estimates. In performing statistical analyses, sample weights (_FINALWT), provided by the BRFSS, were used to calculate representative population-based estimates and to adjust for non-telephone coverage areas and non-response associated with telephone surveys.

We examined obesity and F/V consumption as response variables. For obesity, BRFSS respondents provided information about their height and weight from which their body mass index (BMI) was computed. BMI was calculated as weight in kilograms divided by height in meters squared (kg/m^2^), and respondents whose BMI was over 30 kg/m^2 ^were considered obese. For F/V consumption, the BRFSS variable _FRTINDX was used. The index was derived from the six questions of servings per day F/V consumption: (1) "How often do you drink fruit juice such as orange, grapefruit, or tomato?", (2) "Not counting juice, how often do you eat fruit?", (3) "How often do you eat green salad?", (4) "How often do you eat potatoes not including French fries, fried potatoes, or potato chips?", (5) "How often do you eat carrots?", and (6) "Not counting carrots, potatoes, or salad, how many servings of vegetables do you usually eat? (Example: A serving of vegetables at both lunch and dinner would be two servings.)" Respondents were classified into those who had five or more servings per day versus less than five. The F/V consumption variable was not uniformly available in all states for 2001, 2004, and 2006, thus this variable was analyzed using only the four available years (2000, 2002, 2003, and 2005).

The sample sizes for obesity and F/V consumption variables were 1,477,828 and 836,281, respectively, after excluding missing values and non-responses. Imputation to replace missing data was not considered because of the large amount of missing data on F/V in 2001, 2004, and 2006. About 4.5% of all respondents did not provide information on their height and weight and nearly 80% of these respondents were female in both metro and nonmetro counties. There was, however, no difference between height/weight respondents and non-respondents in F/V consumption in both metro and nonmetro counties, and other demographic characteristics were similar. The percent of respondents who did not provide information on their F/V consumption in the selected four years was 1.6% in metro and 1.0% in nonmetro counties. The difference between F/V respondents and non-respondents in obesity prevalence was small in both metro and nonmetro counties, and other demographic characteristics were similar.

Data on supermarkets were obtained from the 2006 Census Zip Code Business Pattern (ZBP) classified by the North American Industry Classification System (NAICS). Two NAICS codes (445110 and 452910) were combined to form a single supermarket and supercenter category (hereafter referred to as supermarkets) and georeferenced at the ZIP Code level. These two NAICS codes classified supermarkets and other grocery stores (except convenience stores), and warehouse clubs and supercenters, respectively. They were selected because both types of establishments primarily engaged in retailing a general line of food and groceries including fresh fruit and vegetables.

The NAICS data were reported in several classes based on the number of employees. The classes were 1-4, 5-9, 10-19, 20-49, 50-99, 100-249, 250-499, 500-999, and 1,000 or more employees. To take into account potential store size effects, the number of employees was pooled to create small, medium, and large supermarket categories, which were based on 1-19, 20-49, and 50 or more employees, respectively. The breakdown of NAICS data is provided in Table 1. In general, smaller supermarkets are more numerous than medium and larger stores. Although there is no direct way to compare the number of employees with store volume, the number of employees, as a surrogate for store size, indicates the scale of operation and these two factors should be positively correlated [47]. Given this assumption, larger supermarkets generally carry a wide variety of healthy food items at a lower cost than smaller supermarkets [39,48]. In previous work, supermarkets were differentiated from smaller stores based on chain names or annual payroll of greater than 50 employees [22,40,47], and the smallest unit of employment size class is generally recognized as less than 20 employees in economic research [49,50].

**Table 1:** Number of supermarkets (NAICS 445110) and supercenters (NAICS 452910) and percent by size categories

Number of Employee	NAICS 445110	%	NAICS 452910	%	2 Codes Combined	%
Small						
1-4	24639		70		24709	
5-9	7487		15		7502	
10-19	6695		15		6710	
Subtotal	38821	58.8	100	3.1	38921	56.2
Medium						
20-49	9493	14.4	62	1.9	9555	13.8
Large						
50-99	9401		103		9504	
100-249	7816		1092		8908	
250-499	494		1509		2003	
500-999	26		344		370	
1000+	3		0		3	
Subtotal	17740	26.9	3048	95.0	20788	30.0
						
Grand total	66054	100	3210	100	69264	100

### Population-weighted mean distance to supermarket

**Figure 1:**
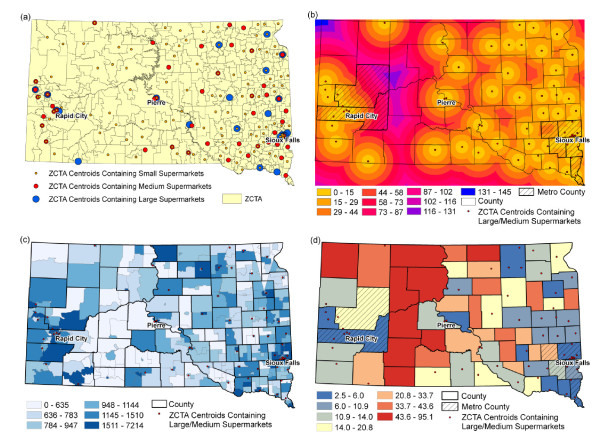
**Computing population-weighted mean distance to supermarket**. (a) Population-weighted ZCTA centroids containing different sizes of supermarkets in South Dakota; (b) Zonal statistics showing mean distance (km) from ZCTA centroids containing large or medium supermarkets in South Dakota; (c) Census 2000 block group population for South Dakota and population-weighted ZCTA centroids containing large or medium supermarkets in South Dakota; and (d) Population-weighted mean distance (km) to large or medium supermarkets in South Dakota by county

Because BRFSS data were not available at finer spatial resolutions than counties, supermarket distances were summarized at the county level. We computed the population-weighted mean distance to supermarket for each county. We obtained population-weighted ZIP Code Tabulation Area (ZCTA) centroids for the entire United States from the Missouri Census Data Center website at http://mcdc.missouri.edu/pub/data/georef/ and joined the NAICS ZIP code data at the national level with three supermarket size categories to the ZCTA centroids. Here, we demonstrate how population-weighted mean distance to supermarket is calculated using South Dakota as an example. The ZCTA centroids that contained different sizes of supermarkets in South Dakota are shown in Figure 1(a). Large cities, such as Sioux Falls and Rapid City, had all three sizes of supermarkets, while smaller cities often contained only small- to medium-sized supermarkets, particularly in sparsely populated areas of the western central South Dakota. Some ZCTA centroids in remote communities, however, contained no supermarkets of any size.

A 1,000 meter resolution raster dataset of Euclidian distance to the nearest supermarket was generated. We used Euclidian distance as a computationally efficient alternative to street network distance because of our focus at the national level. The distance surface to the nearest ZCTA centroid containing either large or medium supermarkets in South Dakota is shown in Figure 1(b). Eastern South Dakota and areas near large cities had shorter mean distance to large or medium supermarkets, compared to the western central part of the state. Zonal statistics were used to compute the mean distance to ZCTA centroids that contained supermarkets for each Census block group (CBG).

The 2000 CBG population data were used as weights and the data for South Dakota are shown in Figure 1(c). The population-weighted mean distance to supermarket for each county was calculated using the following formula:

$$D_{j}=\frac{\Sigma \left(D_{ij}*P_{ij}\right)}{\Sigma P_{ij}}$$

where *D_j _*is the population-weighted mean distance to supermarket for county *j*, *D_ij _*is the mean distance to supermarket in CBG *i*, *P_ij _*is the population in CBG *i*. Summations were across all CBGs within county *j*. The population-weighted mean distances to large or medium supermarkets for all counties in South Dakota are shown in Figure 1(d). Counties in eastern South Dakota and counties with large cities had shorter distances to large or medium supermarkets, while counties in the west-central part of the state had longer distances.

Population-weighted means based on CBG population were used to take into account spatially heterogeneous populations within counties. For example, most of the population of Pennington County lives in Rapid City, whereas the eastern portion of the county has a much lower population density (Figure 1c). The population-weighted mean distance to supermarkets accounts for the fact that populations tend to be concentrated in cities and towns where supermarkets are also located. The population-weighted mean distance is thus typically shorter than the unweighted mean distance (5.6 km versus 6.8 km in the case of Pennington County.)

**Figure 2:**
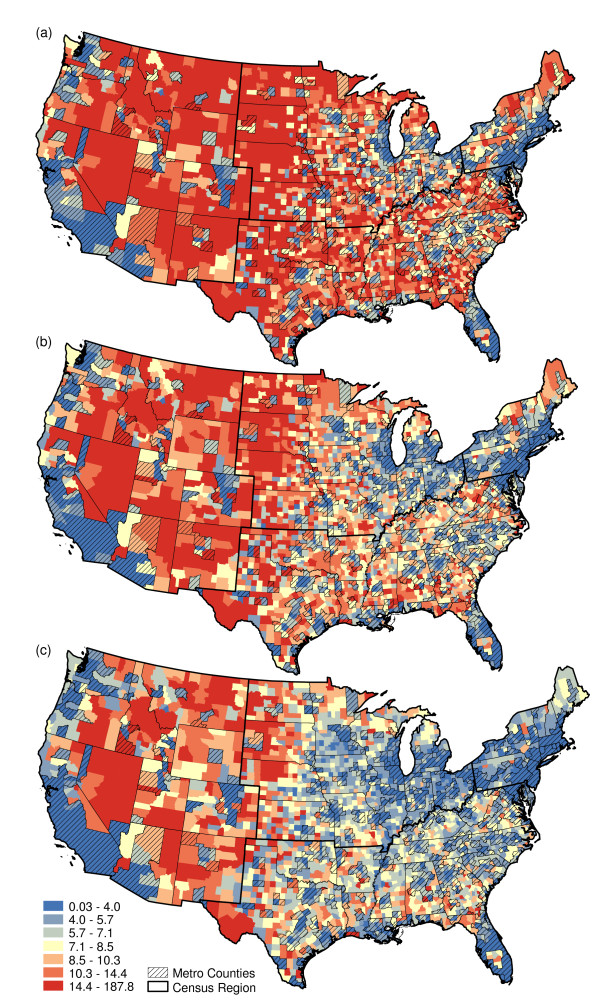
**Population-weighted mean distance (km)**. (a) Large supermarkets, (b) large or medium supermarkets, and (c) large, medium, or small supermarkets (Class intervals are fixed based on map (b) with quantiles).

We repeated these steps to map the population-weighted mean distance to supermarket at the national level for each of three supermarket size categories: large supermarkets; large and medium supermarkets combined; and large, medium, and small supermarkets combined (Figure 2). Large supermarkets were the least numerous, thus, distance to large supermarkets was longer than the other categories, particularly in nonmetropolitan counties. Small and medium supermarkets were more numerous than large supermarkets. Thus, distances to supermarkets decreased when the medium and small store size categories were added in new locations where large supermarkets were absent (see example in Figure 1(a) for locations of supermarket by size).

### Logistic regression models

We developed a series of logistic regression models for obesity and F/V consumption as response variables. We ran the models for metropolitan and nonmetropolitan areas of the United States separately. A metropolitan area is based on urbanized areas of 50,000 or more population within a county or a group of counties. Metropolitan areas consist of core urban area counties along with adjacent counties that have a high degree of social and economic integration measured by commuting ties [51]. In 2000, about 83% of Americans lived in metropolitan areas as defined by the Office of Management and Budget [52]. Nonmetropolitan areas included micropolitan counties with urban clusters of at least 10,000 but less than 50,000, and more sparsely populated noncore counties.

From the BRFSS data, age, sex, race/ethnicity, education, and income levels were included to control for individual level characteristics. Age was grouped into seven categories by 10 year increments, except for the youngest and oldest age cohorts which were 18 to 24 years and 75 years and over. The youngest age cohort was treated as a reference group. For sex, female was treated as a reference group. For race/ethnicity, we included non-Hispanic white, non-Hispanic black, non-Hispanic Asian, Hispanic, American Indian (or Alaskan Native), and included all other races in a separate category as other. Non-Hispanic white was treated as a reference group. Education included four levels: less than high school, high school graduate, some college or technical school, and college graduate. Less than high school was treated as a reference group. Annual household income was grouped into five classes from the original eight classes so that each new class had approximately equal number of respondents. The five classes were less than $15,000, $15,000 to $24,999, $25,000 to $49,999, $50,000 to $74,999 and over $75,000. Less than $15,000 was treated as a reference group. The individual-level BRFSS variables were summarized and compared for metropolitan and nonmetropolitan areas (Table 2).

**Table 2:** Descriptive statistics of sample population with 95% confidence intervals, BRFSS 2000-2006

	Metro (n = 1,220,025)	Nonmetro (n = 552,787)
Age (≥ 18 years)		
Mean (SE)	44.9 (0.03)	47.1 (0.05)
Sex, %		
Male	48.4 [48.2, 48.6]	48.1 [47.8, 48.3]
Female	51.6 [51.4, 51.8]	51.9 [51.7, 52.2]
Race/ethnicity, %		
White	68.6 [68.4, 68.8]	83.0 [82.8, 83.2]
Black	10.4 [10.3, 10.5]	6.5 [6.4, 6.6]
Hispanic	12.4 [12.3, 12.6]	5.0 [4.9, 5.2]
Asian	3.7 [3.6, 3.8]	0.8 [0.7, 0.9]
American Indian	1.2 [1.1, 1.3]	1.7 [1.7, 1.8]
Other	3.6 [3.5, 3.7]	2.9 [2.8, 2.9]
Education, %		
< High school	11.6 [11.4, 11.7]	14.9 [14.7, 15.1]
High school	28.3 [28.2, 28.5]	38.5 [38.3, 38.8]
Some college	27.0 [26.8, 27.1]	25.9 [25.6, 26.1]
College graduate	32.9 [32.8, 33.1]	20.6 [20.4, 20.8]
No response	0.2 [0.2, 0.3]	0.1 [0.1, 0.2]
Income, %		
< $15,000	8.9 [8.8, 9.1]	11.2 [11.1, 11.4]
$15,000-$24,999	14.2 [14.1, 14.3]	18.8 [18.6, 19.0]
$25,000-$49,999	26.0 [25.9, 26.2]	31.2 [30.9, 31.4]
$50,000-$74,999	15.6 [15.5, 15.7]	13.8 [13.6, 13.9]
≥ $75,000	22.4 [22.2, 22.5]	11.0 [10.8, 11.1]
No response	12.9 [12.8, 13.0]	14.0 [13.8, 14.3]
Obesity, %		
BMI ≥ 30 kg/m^2^	21.3 [21.2, 21.4]	24.6 [24.4, 24.8]
F/V consumption, %		
≥ 5 servings per day	24.2 [24.0, 24.3]	21.5 [21.2, 21.7]

Note: Mean and proportions were weighted. If confidence intervals do not overlap between metropolitan and nonmetropolitan areas, percentages are different. Sample size for F/V consumption in metropolitan and nonmetro counties was 665,410 and 314,505, respectively.

**Figure 3:**
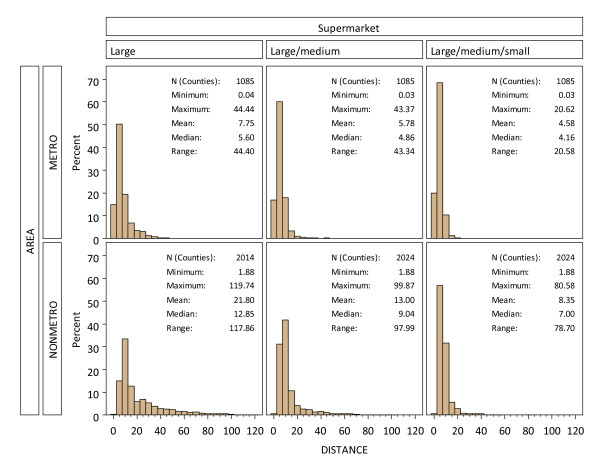
**Histograms of population-weighted mean distance (km) to supermarket by size and metropolitan and nonmetropolitan areas**. (Note: distances greater than 120 km are truncated in the large nonmetro category.)

Population-weighted mean distance to supermarket was included as a county-level metric of variability in supermarket accessibility. Because of the strong positive skew of the supermarket distance variables (Figure 3), we transformed these distance variables using a common (base 10) logarithm. We also included geographic variables using the four Census regions (Northeast, Midwest, South, and West) to control for other unmeasured sources of regional variability unrelated to supermarket distance. The Northeast was selected as a reference group. Odds ratio (OR) estimates and 95% confidence intervals (CI) of the individual- and county-level variables were summarized for each model.

In performing all statistical analyses, sample weights (_FINALWT) provided by the BRFSS were used to obtain representative population-based estimates. The SURVEYMEANS and SURVEYFREQ procedures were used to compute descriptive summaries of obesity, F/V consumption, and population characteristics for metropolitan and nonmetropolitan areas [53]. Because independent variables were measured at two different levels (demographic characteristics at the individual level and supermarket distance and region at the county level) we used a generalized linear model for logistic regression, wherein counties were specified as clusters to account for correlations among individuals sampled within each county. Models were fitted using the GENMOD procedure, which used generalized estimating equations to account for the correlated data structure. All data processing and analyses were carried out using SAS version 9.2. All maps were created using ArcGIS Version 9.3.

## Results

Differences between metropolitan and nonmetropolitan areas are described in Table 2. Overall, obesity was higher in nonmetropolitan areas than in metropolitan areas. F/V consumption was higher in metropolitan areas than in nonmetropolitan areas. Although non-Hispanic white was the majority of residents in both metropolitan and nonmetropolitan areas, there were larger proportions of non-Hispanic black and Hispanic populations in metropolitan areas. In addition, residents living in metropolitan areas were younger and had higher socioeconomic status compared to those in nonmetropolitan areas. Percent of residents who did not complete high school was higher in nonmetropolitan areas, whereas percent of college graduates was higher in metropolitan areas. Percent of residents with household income over $50,000 was higher in metropolitan areas, whereas percent of lower income (< $25,000) was higher in nonmetropolitan areas. There was little difference in sex ratio between metropolitan and nonmetropolitan areas.

ORs for socioeconomic variables, regions, and distance to supermarket were similar for all three supermarket size categories in both the obesity and F/V consumption models. Therefore, only results for the large supermarket class are presented in the text, and additional results for large/medium, and large/medium/small supermarkets are provided in the additional files (see Additional File 1, table S1 and Additional File 2. table S2).

### Distance to supermarket

Metropolitan areas had overall shorter distances to supermarkets for all three store size categories (Figure 3), reflecting higher population densities in metropolitan areas. Nonmetropolitan areas, in contrast, had longer distances to supermarkets, but distances diminished in some areas as more stores of smaller sizes were included (Figure 2c). Distance to supermarket in suburban and exurban counties of the East Coast and Great Lakes regions was particularly sensitive to the addition of smaller supermarkets (Figure 2c compared to Figure 2a). Nonmetropolitan areas of the Great Plains and West, however, still had relatively long distances to supermarkets even if smaller supermarkets were included.

### Obesity - metropolitan area model

The model for obesity in metropolitan areas indicated increased risk of obesity among all age groups compared to the reference group (aged 18-24) (Table 3). The middle-aged cohorts (aged 55-64 and 45-54), however, had larger ORs than other age groups. Males were more likely to be obese, compared to females. Compared to non-Hispanic white, non-Hispanic black was at greater risk of obesity. Education and household income both showed risk reduction for obesity as these socioeconomic variables increased. For geographic regions, the Midwest had a positive association with obesity, compared to the Northeast. Distance to large supermarket was positively associated with the prevalence of obesity in the metropolitan area model. This relationship was also statistically significant for large and medium supermarkets combined and for large, medium, and small supermarkets combined (Additional File 1. Appendix 1).

**Table 3:** Odds ratios for obesity by metro status with distance to large supermarket

	Metro (n = 1,019,585)	Nonmetro (n = 458,243)
Age, 18-24 years (ref.)		
25-34	1.94 [1.86, 2.03]**	1.92 [1.80, 2.06]**
35-44	2.41 [2.32, 2.51]**	2.30 [2.15, 2.47]**
45-54	2.89 [2.77, 3.02]**	2.60 [2.43, 2.77]**
55-64	3.00 [2.87, 3.13]**	2.58 [2.42, 2.76]**
65-74	2.24 [2.14, 2.34]**	1.94 [1.81, 2.08]**
≥ 75.	1.28 [1.22, 1.35]**	1.02 [0.95, 1.10]
Sex, Female (ref.)		
Male	1.09 [1.06, 1.12]**	1.05 [1.02, 1.07]**
Race/ethnicity, White (ref.)		
Black	1.79 [1.73, 1.85]**	1.70 [1.62, 1.78]**
Hispanic	1.23 [1.17, 1.30]**	1.21 [1.12, 1.30]**
Asian	0.54 [0.49, 0.60]**	0.56 [0.44, 0.71]**
American Indian	1.31 [1.18, 1.45]**	1.46 [1.31, 1.62]**
Other	1.03 [0.96, 1.09]	1.17 [1.09, 1.27]**
Education, < High school (ref.)		
H.S.	0.88 [0.85, 0.91]**	0.92 [0.89, 0.96]**
Some college	0.86 [0.83, 0.90]**	0.92 [0.88, 0.96]**
College graduate	0.60 [0.57, 0.62]**	0.73 [0.69, 0.77]**
Income, < $15,000 (ref.)		
$15,000-$24,999	0.91 [0.88, 0.94]**	0.90 [0.86, 0.94]**
$25,000-$49,999	0.83 [0.80, 0.86]**	0.79 [0.75, 0.82]**
$50,000-$74,999	0.79 [0.76, 0.82]**	0.73 [0.70, 0.77]**
≥ $75,000	0.66 [0.63, 0.69]**	0.60 [0.57, 0.64]**
Region, Northeast (ref.)		
Midwest	1.10 [1.06, 1.15]**	1.09 [1.04, 1.15]**
South	1.00 [0.96, 1.05]	1.07 [1.02, 1.13]**
West	0.98 [0.93, 1.02]	0.87 [0.81, 0.93]**
Supermarket distance, log10		
Large supermarket	1.24 [1.19, 1.29]**	1.00 [0.94, 1.06]

Note: Numbers in brackets are 95% confidence intervals for odds ratios.

* *p *< 0.05, ** *p *< 0.01

### Obesity - nonmetropolitan area model

The model for obesity in nonmetropolitan counties was similar to the metropolitan area model with higher ORs occurring among the middle-age cohorts (aged 45-54 and 55-64) compared to the reference group (Table 3). The ORs for sex, race/ethnicity, education, and income in the nonmetropolitan area model were generally similar to the metropolitan area model. Obesity risk in the Midwest and South in the nonmetropolitan area model was greater than in the Northeast. Obesity risk in the West, on the contrary, was less than in the Northeast. Unlike metropolitan areas, distance to supermarket was not associated with obesity prevalence for any of the supermarket size categories (Additional File 1. Appendix 1).

### F/V consumption - metropolitan area model

Compared to the reference group, intermediate age cohorts (aged 25-34, 35-44, and 45-54) generally had a lower odds of consuming F/V, but the odds increased with age thereafter (Table 4). Females were more likely to eat F/V, compared to males. For race/ethnicity, all racial/ethnic groups were more likely to consume F/V, compared to non-Hispanic white. Increases in educational and household income levels had positive relationships with consumption of F/V. The Midwest and South had lower F/V consumption compared to the Northeast. Distance to large supermarket was negatively associated with the consumption of F/V in the metropolitan area model. This relationship was also statistically significant for large and medium supermarkets combined, but not for large, medium, and small supermarkets combined (Additional File 2. Appendix 2).

**Table 4:** Odds ratios for fruit and vegetable consumption by metro status with distance to large supermarket

	Metro (n = 568,584)	Nonmetro (n = 267,697)
Age, 18-24 years (ref.)		
25-34	0.81 [0.77, 0.84]**	0.81 [0.75, 0.88]**
35-44	0.85 0.81, 0.89]**	0.84 [0.77, 0.91]**
45-54	0.94 [0.90, 0.98]**	0.95 [0.88, 1.03]
55-64	1.09 [1.04, 1.15]**	1.18 [1.09, 1.28]**
65-74	1.41 [1.33, 1.49]**	1.58 [1.45, 1.72]**
≥ 75	1.84 [1.71, 1.97]**	1.99 [1.82, 2.18]**
Sex, Female (ref.)		
Male	0.59 [0.58, 0.61]**	0.59 [0.56, 0.61]**
Race/ethnicity, White (ref.)		
Black	1.08 [1.04, 1.12]**	0.95 [0.88, 1.03]
Hispanic	1.17 [1.12, 1.23]**	1.18 [1.05, 1.31]**
Asian	1.26 [1.16, 1.37]**	1.22 [0.99, 1.51]
American Indian	1.38 [1.18, 1.62]**	1.36 [1.14, 1.62]**
Other	1.34 [1.26, 1.43]**	1.24 [1.10, 1.40]**
Education, < High school (ref.)		
H.S.	1.06 [1.00, 1.11]*	1.09 [1.02, 1.17]**
Some college	1.30 [1.22, 1.39]**	1.48 [1.38, 1.59]**
College grad	1.70 [1.61, 1.80]**	1.93 [1.80, 2.08]**
Income, < $15,000 (ref.)		
$15,000-$24,999	1.06 [1.01, 1.12]*	1.16 [1.09, 1.23]**
$25,000-$49,999	1.08 [1.01, 1.15]*	1.18 [1.10, 1.26]**
$50,000-$74,999	1.12 [1.04, 1.19]**	1.29 [1.20, 1.39]**
≥ $75,000	1.25 [1.16, 1.35]**	1.43 [1.33, 1.55]**
Region, Northeast (ref)		
Midwest	0.80 [0.77, 0.83]**	0.83 [0.77, 0.89]**
South	0.90 [0.86, 0.94]**	0.89 [0.83, 0.95]**
West	0.98 [0.93, 1.03]	0.96 [0.88, 1.04]
Supermarket distance, log10		
Large supermarket	0.95 [0.92, 0.99]*	0.99 [0.91, 1.06]

Note: Numbers in brackets are 95% confidence intervals for odds ratios.

* *p *< 0.05, ** *p *< 0.01

### F/V consumption - nonmetropolitan area model

The associations of individual-level covariates with F/V consumption in the nonmetropolitan area models were similar to those in the metropolitan area models (Table 4). In the nonmetropolitan area model, the middle-age cohort (aged 45-54) was not significantly different from the reference group. In addition, the odds of consuming F/V among non-Hispanic black and non-Hispanic Asian was not significantly different from that of non-Hispanic white. The higher education and income groups had greater odds of consuming F/V, compared to the reference group. The Midwest and South had lower F/V consumption, compared to the Northeast. Distance to supermarket was not associated with the prevalence of F/V consumption for any size categories in the nonmetropolitan area models (Additional File 2. Appendix 2).

## Discussion

These results support the idea that travel distance to supermarkets is associated with obesity and fruit and vegetable intake in adults, but they highlight important differences in these relationships between metropolitan and nonmetropolitan areas. We found that distance to supermarket had positive associations with obesity and negative associations with fruit and vegetable consumption in metropolitan areas, but not in nonmetropolitan areas. The prevalence of obesity was higher in nonmetropolitan areas than in metropolitan areas, and this fact may be in part due to the longer distances to supermarkets in nonmetropolitan areas.

Although obesity and fruit and vegetable consumption were analyzed separately, the increased likelihood of obesity with longer distance to supermarket that was seen in metropolitan areas may be related to the decreased likelihood of consuming fruit and vegetables with longer distance to supermarket. Our findings indicated, however, that distance to supermarket was not associated with either obesity or fruit and vegetable consumption in nonmetropolitan areas. Physical distance to reach supermarkets likely has a different impact in metropolitan and nonmetropolitan areas. For example, traveling for a few miles may take longer in congested urban areas than in rural areas with less traffic. Thus, the role of supermarket accessibility measured by distance in the food environment appears to be fundamentally different in urban- versus rural-dominated environments.

One possible explanation for the lack of association of supermarket distance with obesity and fruit and vegetable consumption in nonmetropolitan areas is differences in transportation infrastructure and travel behavior compared to metropolitan areas. Inner-city residents make the most trips, but their trips have the shortest durations and they spend the least time in travel, whereas residents of commuter belt areas beyond the suburbs make fewer trips, but their trips have the longest durations and they spend the most time in travel [54]. Similarly, rural populations travel further and spend more time travelling for medical care than urban populations do, and distance traveled and time spent in travel are inversely related to population density [55]. These differences could explain the higher obesity and lower fruit and vegetable consumption in nonmetropolitan areas than in metropolitan areas if the residents of these rural areas make fewer trips to supermarkets, and as a result, purchase more processed foods and fewer perishable food items elsewhere as alternative diets.

Because of limited public transportation, rural residents rely on private automobiles for most travel needs, regardless of age, race, and income, whereas urban and suburban residents with good access to public transportation and mixed land use have lower automobile ownership [54,56-58]. Despite having to drive long distances, rural residents may not vary greatly in their ability to reach various types of services, including grocery shopping. Private automobiles allow rural residents to travel to reach services according to their own schedules. Variability in distance to supermarkets in nonmetropolitan areas, therefore, may not translate into differences in higher obesity prevalence and lower fruit and vegetable consumption. In contrast, supermarket distance may have a greater impact on food shopping behavior in metropolitan areas if urban residents do not own private automobiles and are dependent on public transportation. For example, if supermarkets are scarce in low-income urban neighborhoods, then residents of these neighborhoods would have less access to healthy foods if they cannot reach supermarkets while stores are open.

In many nonmetropolitan areas, residents may have limited accessibility to healthy food, but accessing supermarkets may not impose a greater burden if communities make efforts to change the local food infrastructures. Some unique characteristics of nonmetropolitan areas may contribute to the weaker association of distance to supermarkets with obesity and healthy food consumption. For example, availability of alternative local sources of fruit and vegetables through nonconventional food-selling environments, such as farmer's markets, fruit and vegetable stands, home gardens, and general merchandise stores, may reduce the importance of supermarket accessibility in determining diet and health [31,59]. In addition, direct exchange of food among rural residents through personal connections and community social capital, such as distributing foods through rural churches and community centers, has been reported in some areas where few or no grocery stores exist [41,60]. Furthermore, an important element of the urban food desert concept is that decreased access to supermarkets and other sources of healthy food occurs in conjunction with increased access to convenience stores, fast-food restaurants, and other sources of unhealthy foods [19-21]. It may be that in many nonmetropolitan areas, residents have limited accessibility to all sources of food, and accessing supermarkets is not more difficult than accessing alternative sources of unhealthy food.

Another important assumption of supermarket accessibility research is that larger supermarkets provide a more extensive and affordable selection of healthy foods than smaller supermarkets [18]. Although we examined three-size classes of supermarkets, we found no store size effects on the prevalence of obesity in either the metropolitan or nonmetropolitan area models, as well as for F/V consumption, except for the large/medium/small supermarkets combined model. However, we did not directly quantify food selections or prices in these three sizes of supermarkets. If supermarkets in nonmetropolitan areas do not provide as wide a selection of fruit and vegetables as supermarkets in metropolitan areas, the supermarkets may have a weaker relationship to the food environment in nonmetropolitan areas.

The significant positive association of obesity with distance to supermarket in metropolitan areas may also reflect the indirect impacts of suburban sprawl. Within metropolitan areas, urban-dominated counties in the core central cities typically had shorter distances to supermarkets, whereas outlying counties had longer distances. Newer suburban housing developments are typically oriented toward automobile travel, which leads to the increased risk of obesity due to limited connectivity and walkability between residential and commercial locations [61-63]. At the metropolitan area level, an index of urban sprawl was a significant predictor for the risk of being overweight and obese among adults [64]. In addition, there was an association between travel distance by automobiles and obesity in California [65] and each additional hour spent in a car per day was also associated with increase in the likelihood of obesity in Georgia [66]. These studies provide some evidence that suburban sprawl forces people to travel by automobiles and it discourages physical activity, which in turn, contributes to the increased risk of obesity. Our findings in the metropolitan area models are consistent with such suburban sprawl hypotheses if the distance to supermarket is considered a proxy for time spent in a car.

### Caveats and limitation

Analyses using data drawn from telephone surveys may be subject to bias because BMI and F/V consumption measures in the BRFSS are based on individuals' recall. Studies showed that population-level bias in self-reported height and weight was larger in telephone surveys, compared to in-person interviews that involved physical examinations. BRFSS underestimates the overall prevalence of overweight and obesity, and this bias could be attributed to such survey mode effects [67,68]. Our study, however, focused on studying the correlates of spatial variability in obesity rather than precisely quantifying the prevalence of obesity from multiple databases, and these self-reporting biases should not influence our ability to detect these relationships. In addition we only examined one broad metric of healthy food consumption (the number of daily servings of fruit and vegetables). Different results could be obtained based on more detailed measurements on the quality and quantity of healthy food consumption [69].

The advantage of using the BRFSS data for this analysis was the availability of a representative sample covering virtually all counties within the conterminous United States. However, because counties are the finest geographic resolution at which the BRFSS data can be obtained, we had to summarize distance to supermarket as a population-weighted mean at the county level. In our analyses, this statistic was assumed to define the food environment for all individuals within the county. In reality, most counties encompass a mixture of neighborhoods with varying scales of supermarket accessibility, and this variability is not captured by our county-level indices. Different results could, therefore, be obtained by more localized analyses accounting for neighborhood and household effects.

Because we obtained supermarket data from the Zip Code Business Patterns dataset classified by the NAICS, we geocoded supermarkets to the population-weighted centroid of each ZCTA. Population-weighted centroids generally fall within the largest town or a segment of a city in the ZCTA, which is where supermarkets and other large retail stores are most likely to be located. Although we did not have the exact spatial locations of supermarkets, this lack of precision is probably not critical because distances were aggregated at the county level. More detailed, localized studies based on geocoded street addresses of individual residences and supermarkets in rural areas are needed in order to corroborate the results of this study, and provide additional insights into the proximal effects of supermarket accessibility in rural areas.

In addition, industry classification systems may be increasingly irrelevant as local economies become more diversified. Business establishments may involve sector changes, and newly emerging firms may not be recognized by existing classifications [70]. Despite this limitation, a study showed that business listings provided by commercial databases based on the NAICS and direct observations of business establishments were highly compatible at the neighborhood level [71]. Although rural areas may be associated with greater uncertainty for business existence and operations, the NAICS is a standardized business classification used by Federal statistical agencies for analyzing the U.S. economy.

## Conclusions

Obesity prevalence increased and fruit and vegetable consumption decreased with increasing distance to supermarkets in metropolitan areas, but not in nonmetropolitan areas. These relationships in metropolitan areas likely reflect, at least in part, the higher availability of fruit and vegetables in areas with high supermarket accessibility. In contrast, other social and environmental factors besides supermarket accessibility may have stronger relationships with obesity and F/V consumption in nonmetropolitan areas. These include individual-level attributes such as race, income, and education, and also include other factors not considered in this study such as physical activity. Nonmetropolitan areas are also composed of diverse population groups which may have access to healthy foods through other sources besides supermarkets. One important implication of this study is that simple measures of distance to supermarket are not sufficient to characterize the diverse food environment in rural areas. Instead, future studies should attempt to more precisely quantify the availability and affordability of foods in rural environments, and consider alternative sources of healthy foods besides supermarkets.

## Competing interests

The authors declare that they have no competing interests.

## Authors' contributions

MCW and AM conceived and designed the study. AM was responsible for development and management of the databases. Both authors carried out the statistical analyses and contributed to the interpretation of the results and writing of the manuscript. All authors read and approved the final manuscript.

## Supplementary Material

Additional file 1 **Table s1 - Odds ratio for obesity by metro status for each store size category**. This table shows odds ratios for obesity by metropolitan and nonmetropolitan areas for each store size category. Click here for file

Additional file 2 **Table s2 - Odds ratio for fruit and vegetable consumption by metro status for each store size category**. This table shows odds ratios for F/V consumption by metropolitan and nonmetropolitan areas for each store size category. Click here for file
