# Supplementary material for: Associations of supermarket accessibility with obesity and fruit and vegetable consumption in the conterminous United States
Source: Int J Health Geogr. 2010 Oct 8;9:49. doi: 10.1186/1476-072X-9-49 (PMC2959055; doi:10.1186/1476-072X-9-49)
Supplement: Additional file 1 — Table s1 - Odds ratio for obesity by metro status for each store size category. This table shows odds ratios for obesity by metropolitan and nonmetropolitan areas for each store size category. [file 1476-072X-9-49-S1.PDF]

Additional file 1 - Odds ratios for obesity by metro status for each store size category

	Metro			Nonmetro		
	L SM	L/M SM	L/M/S SM	L SM	L/M SM	L/M/S SM
Age, 18-24 years (ref.)						
25-34	1.94 [1.86, 2.03]**	1.94 [1.86, 2.03]**	1.94 [1.86, 2.03]**	1.92 [1.80, 2.06]**	1.92 [1.80, 2.06]**	1.92 [1.80, 2.06]**
35-44	2.41 [2.32, 2.51]**	2.42 [2.32, 2.52]**	2.42 [2.32, 2.52]**	2.30 [2.15, 2.47]**	2.31 [2.15, 2.47]**	2.30 [2.15, 2.47]**
45-54	2.89 [2.77, 3.02]**	2.89 [2.77, 3.02]**	2.89 [2.77, 3.02]**	2.60 [2.43, 2.77]**	2.60 [2.43, 2.77]**	2.60 [2.43, 2.77]**
55-64	3.00 [2.87, 3.13]**	3.00 [2.87, 3.13]**	3.00 [2.87, 3.14]**	2.58 [2.42, 2.76]**	2.59 [2.42, 2.76]**	2.58 [2.42, 2.76]**
65-74	2.24 [2.14, 2.34]**	2.24 [2.14, 2.35]**	2.24 [2.14, 2.35]**	1.94 [1.81, 2.08]**	1.94 [1.81, 2.08]**	1.94 [1.81, 2.08]**
≥ 75	1.28 [1.22, 1.35]**	1.28 [1.22, 1.35]**	1.28 [1.22, 1.35]**	1.02 [0.95, 1.10]	1.02 [0.95, 1.10]	1.02 [0.95, 1.10]
Sex, Female (ref.)						
Male	1.09 [1.06, 1.12]**	1.09 [1.06, 1.12]**	1.09 [1.06, 1.12]**	1.05 [1.02, 1.07]**	1.05 [1.02, 1.07]**	1.05 [1.02, 1.07]**
Race/ethnicity, White (ref.)						
Black	1.79 [1.73, 1.85]**	1.79 [1.73, 1.85]**	1.79 [1.73, 1.84]**	1.70 [1.62, 1.78]**	1.70 [1.62, 1.78]**	1.70 [1.62, 1.78]**
Hispanic	1.23 [1.17, 1.30]**	1.23 [1.17, 1.30]**	1.23 [1.17, 1.30]**	1.21 [1.12, 1.30]**	1.21 [1.12, 1.30]**	1.21 [1.12, 1.30]**
Asian	0.54 [0.49, 0.60]**	0.54 [0.49, 0.60]**	0.54 [0.48, 0.60]**	0.56 [0.44, 0.71]**	0.56 [0.44, 0.71]**	0.56 [0.44, 0.71]**
American Indian	1.31 [1.18, 1.45]**	1.31 [1.18, 1.45]**	1.31 [1.18, 1.45]**	1.46 [1.31, 1.62]**	1.46 [1.32, 1.62]**	1.46 [1.32, 1.62]**
Other	1.03 [0.96, 1.09]	1.03 [0.96, 1.09]	1.03 [0.96, 1.09]	1.17 [1.09, 1.27]**	1.17 [1.09, 1.27]**	1.17 [1.09, 1.27]**
Education, < High school (ref.)						
H.S.	0.88 [0.85, 0.91]**	0.88 [0.85, 0.91]**	0.88 [0.85, 0.91]**	0.92 [0.89, 0.96]**	0.92 [0.89, 0.96]**	0.92 [0.89, 0.96]**
Some college	0.86 [0.83, 0.90]**	0.86 [0.83, 0.90]**	0.86 [0.83, 0.90]**	0.92 [0.88, 0.96]**	0.92 [0.88, 0.96]**	0.92 [0.88, 0.96]**
College graduate	0.60 [0.57, 0.62]**	0.59 [0.57, 0.62]**	0.59 [0.57, 0.62]**	0.73 [0.69, 0.77]**	0.73 [0.69, 0.77]**	0.73 [0.69, 0.77]**
Income, < $15,000 (ref.)						
$15,000-$24,999	0.91 [0.88, 0.94]**	0.91 [0.88, 0.94]**	0.91 [0.88, 0.94]**	0.90 [0.86, 0.94]**	0.90 [0.86, 0.94]**	0.90 [0.86, 0.94]**
$25,000-$49,999	0.83 [0.80, 0.86]**	0.83 [0.80, 0.86]**	0.83 [0.80, 0.86]**	0.79 [0.75, 0.82]**	0.79 [0.75, 0.82]**	0.79 [0.75, 0.82]**
$50,000-$74,999	0.79 [0.76, 0.82]**	0.79 [0.76, 0.82]**	0.79 [0.76, 0.82]**	0.73 [0.70, 0.77]**	0.73 [0.70, 0.77]**	0.73 [0.70, 0.77]**
≥ $75,000	0.66 [0.63, 0.69]**	0.66 [0.63, 0.69]**	0.66 [0.63, 0.69]**	0.60 [0.57, 0.64]**	0.60 [0.57, 0.64]**	0.60 [0.57, 0.64]**
Region, Northeast (ref.)						
Midwest	1.10 [1.06, 1.15]**	1.10 [1.06, 1.15]**	1.10 [1.05, 1.15]**	1.09 [1.04, 1.15]**	1.09 [1.04, 1.15]**	1.09 [1.04, 1.15]**
South	1.00 [0.96, 1.05]	1.00 [0.95, 1.04]	0.99 [0.95, 1.04]	1.07 [1.02, 1.13]**	1.08 [1.02, 1.13]**	1.07 [1.02, 1.13]**
West	0.98 [0.93, 1.02]	0.97 [0.93, 1.01]	0.97 [0.93, 1.01]	0.87 [0.81, 0.93]**	0.88 [0.82, 0.94]**	0.87 [0.81, 0.94]**
SM distance, log10						
L SM	1.24 [1.19, 1.29]**			1.00 [0.94, 1.06]		
L/M SM		1.25 [1.19, 1.30]**			0.96 [0.88, 1.05]	
L/M/S SM			1.25 [1.19, 1.31]**			0.98 [0.88, 1.09]

Note: Numbers in brackets are 95% confidence intervals for odds ratios.

L = large, M = medium, S = small, SM = supermarket

* $p < 0.05$, ** $p < 0.01$
