# Supplementary material for: Associations of supermarket accessibility with obesity and fruit and vegetable consumption in the conterminous United States
Source: Int J Health Geogr. 2010 Oct 8;9:49. doi: 10.1186/1476-072X-9-49 (PMC2959055; doi:10.1186/1476-072X-9-49)
Supplement: Additional file 2 — Table s2 - Odds ratio for fruit and vegetable consumption by metro status for each store size category. This table shows odds ratios for F/V consumption by metropolitan and nonmetropolitan areas for each store size category. [file 1476-072X-9-49-S2.PDF]

Additional file 2 - Odds ratios for fruit and vegetable consumption by metro status for each store size category

	Metro			Nonmetro		
	L SM	L/M SM	L/M/S SM	L SM	L/M SM	L/M/S SM
Age, 18-24 years (ref.)						
25-34	0.81 [0.77, 0.84]**	0.81 [0.77, 0.84]**	0.81 [0.77, 0.84]**	0.81 [0.75, 0.88]**	0.81 [0.75, 0.88]**	0.81 [0.75, 0.88]**
35-44	0.85 [0.81, 0.89]**	0.85 [0.81, 0.89]**	0.85 [0.81, 0.89]**	0.84 [0.77, 0.91]**	0.84 [0.77, 0.91]**	0.84 [0.77, 0.91]**
45-54	0.94 [0.90, 0.98]**	0.94 [0.90, 0.98]**	0.94 [0.90, 0.98]**	0.95 [0.88, 1.03]	0.95 [0.88, 1.03]	0.95 [0.88, 1.03]
55-64	1.09 [1.04, 1.15]**	1.09 [1.04, 1.15]**	1.09 [1.04, 1.15]**	1.18 [1.09, 1.28]**	1.18 [1.09, 1.28]**	1.18 [1.09, 1.28]**
65-74	1.41 [1.33, 1.49]**	1.41 [1.33, 1.49]**	1.41 [1.33, 1.49]**	1.58 [1.45, 1.72]**	1.58 [1.45, 1.72]**	1.58 [1.45, 1.72]**
≥ 75	1.84 [1.71, 1.97]**	1.84 [1.72, 1.97]**	1.84 [1.72, 1.98]**	1.99 [1.82, 2.18]**	1.99 [1.82, 2.18]**	1.99 [1.82, 2.18]**
Sex, Female (ref.)						
Male	0.59 [0.58, 0.61]**	0.59 [0.58, 0.61]**	0.59 [0.58, 0.61]**	0.59 [0.56, 0.61]**	0.59 [0.56, 0.61]**	0.59 [0.56, 0.61]**
Race/ethnicity, White (ref.)						
Black	1.08 [1.04, 1.12]**	1.08 [1.04, 1.12]**	1.08 [1.04, 1.12]**	0.95 [0.88, 1.03]	0.95 [0.88, 1.03]	0.95 [0.88, 1.03]
Hispanic	1.17 [1.12, 1.23]**	1.18 [1.12, 1.23]**	1.18 [1.12, 1.24]**	1.18 [1.05, 1.31]**	1.18 [1.05, 1.31]**	1.18 [1.05, 1.31]**
Asian	1.26 [1.16, 1.37]**	1.26 [1.16, 1.37]**	1.26 [1.16, 1.37]**	1.22 [0.99, 1.51]	1.22 [0.99, 1.51]	1.22 [0.99, 1.51]
American Indian	1.38 [1.18, 1.62]**	1.38 [1.18, 1.62]**	1.39 [1.18, 1.62]**	1.36 [1.14, 1.62]**	1.36 [1.14, 1.62]**	1.36 [1.14, 1.62]**
Other	1.34 [1.26, 1.43]**	1.34 [1.26, 1.43]**	1.34 [1.26, 1.43]**	1.24 [1.10, 1.40]**	1.24 [1.10, 1.40]**	1.24 [1.10, 1.40]**
Education, < High school (ref.)						
H.S.	1.06 [1.00, 1.11]*	1.06 [1.00, 1.11]*	1.06 [1.00, 1.11]*	1.09 [1.02, 1.17]**	1.09 [1.02, 1.17]**	1.09 [1.02, 1.17]**
Some college	1.30 [1.22, 1.39]**	1.30 [1.22, 1.39]**	1.30 [1.22, 1.39]**	1.48 [1.38, 1.59]**	1.48 [1.38, 1.59]**	1.48 [1.38, 1.59]**
College graduate	1.70 [1.61, 1.80]**	1.70 [1.61, 1.80]**	1.70 [1.61, 1.80]**	1.93 [1.80, 2.08]**	1.93 [1.80, 2.08]**	1.93 [1.80, 2.08]**
Income, < $15,000 (ref.)						
$15,000-$24,999	1.06 [1.01, 1.12]*	1.06 [1.01, 1.12]*	1.06 [1.01, 1.12]*	1.16 [1.09, 1.23]**	1.16 [1.09, 1.23]**	1.16 [1.09, 1.23]**
$25,000-$49,999	1.08 [1.01, 1.15]*	1.08 [1.01, 1.15]*	1.08 [1.01, 1.15]*	1.18 [1.10, 1.26]**	1.18 [1.10, 1.26]**	1.18 [1.10, 1.26]**
$50,000-$74,999	1.12 [1.04, 1.19]**	1.11 [1.04, 1.19]**	1.12 [1.04, 1.19]**	1.29 [1.20, 1.39]**	1.29 [1.20, 1.39]**	1.29 [1.20, 1.39]**
≥ $75,000	1.25 [1.16, 1.35]**	1.25 [1.16, 1.35]**	1.25 [1.16, 1.35]**	1.43 [1.33, 1.55]**	1.44 [1.33, 1.55]**	1.43 [1.33, 1.55]**
Region, Northeast (ref.)						
Midwest	0.80 [0.77, 0.83]**	0.80 [0.77, 0.83]**	0.80 [0.77, 0.83]**	0.83 [0.77, 0.89]**	0.83 [0.77, 0.89]**	0.83 [0.77, 0.89]**
South	0.90 [0.86, 0.94]**	0.90 [0.86, 0.94]**	0.90 [0.86, 0.94]**	0.89 [0.83, 0.95]**	0.89 [0.83, 0.95]**	0.89 [0.83, 0.96]**
West	0.98 [0.93, 1.03]	0.98 [0.93, 1.03]	0.98 [0.93, 1.03]	0.96 [0.88, 1.04]	0.96 [0.88, 1.04]	0.96 [0.88, 1.05]
SM distance, log10						
L SM	0.95 [0.92, 0.99]*			0.99 [0.91, 1.06]		
L/M SM		0.96 [0.92, 1.00]*			0.99 [0.88, 1.11]	
L/M/S SM			0.96 [0.92, 1.00]			0.97 [0.84, 1.12]

Note: Numbers in brackets are 95% confidence intervals for odds ratios.

L = large, M = medium, S = small, SM = supermarket

* $p < 0.05$, ** $p < 0.01$
